# Effectiveness and Safety of Setmelanotide in a Patient With a Heterozygous *PCSK1* Deficiency

**DOI:** 10.1002/oby.70213

**Published:** 2026-05-01

**Authors:** Ellina Lytvyak, Arshdeep Rattol, Eduardo Grunvald

**Affiliations:** ^1^ Department of Medicine University of Alberta Edmonton Alberta Canada; ^2^ School of Medicine University of California San Diego La Jolla California USA

**Keywords:** c.661A>G (p.Asn221Asp) variant, effectiveness, heterozygous PCSK1 deficiency, obesity, safety, setmelanotide, weight loss

## Abstract

Setmelanotide, a melanocortin 4 receptor (MC4R) agonist, is a promising pharmacological treatment option for people with rare monogenic obesity conditions affecting the leptin‐melanocortin signaling pathway, including proprotein convertase subtilisin/kexin type 1 (*PCSK1*) gene mutations. It has been studied in people with homozygous mutations causing a complete deficiency of *PCSK1*. We report the first case of a 40‐year‐old female with a heterozygous *PCSK1* N221D (c.661 A>G) variant mutation leading to severe early‐onset treatment‐resistant obesity with previous suboptimal response to bariatric surgery and conventional obesity medications, who achieved a total weight loss of 11.8% with setmelanotide treatment over the course of 3 months. Although her mutation confers a loss of 10% to 30% enzymatic function in in vitro studies, setmelanotide was highly effective in treating her obesity. It is also the first reported case of a cutaneous adverse effect of setmelanotide in the form of severe skin hyperpigmentation in a patient with a pathogenic *PCSK1* variant. This case underscores the effectiveness and safety of setmelanotide in a heterozygous *PCSK1* mutation.

**Abbreviations**
HbA1C: hemoglobin A1C
HDL: high‐density lipoprotein
LDL: low‐density lipoprotein
LEPR: leptin receptor
MC1R: melanocortin 1 receptor
MC4R: melanocortin 4 receptor
PC1/3: proprotein convertase 1/3
PCSK1: proprotein convertase subtilisin/kexin type 1
POMC: pro‐opiomelanocortin

## 1 Introduction

Proprotein convertase subtilisin/kexin type 1 (*PCSK1*) deficiency, often caused by homo‐ or compound heterozygous mutations, is a rare genetic obesity condition currently reported in fewer than 50 individuals worldwide [1, 2]. Pathogenic variants of *PCSK1*, including c.661A>G (p.Asn221Asp), can act as deleterious mutations leading to reduced catalytic activity of proprotein convertase 1/3 (PC1/3) and subsequent disruption of the leptin‐melanocortin signaling pathway [3]. The resulting dysregulation of energy homeostasis, metabolism, and appetite regulation can independently cause early‐onset obesity and hyperphagia [2, 3, 4, 5].

**FIGURE 1:**
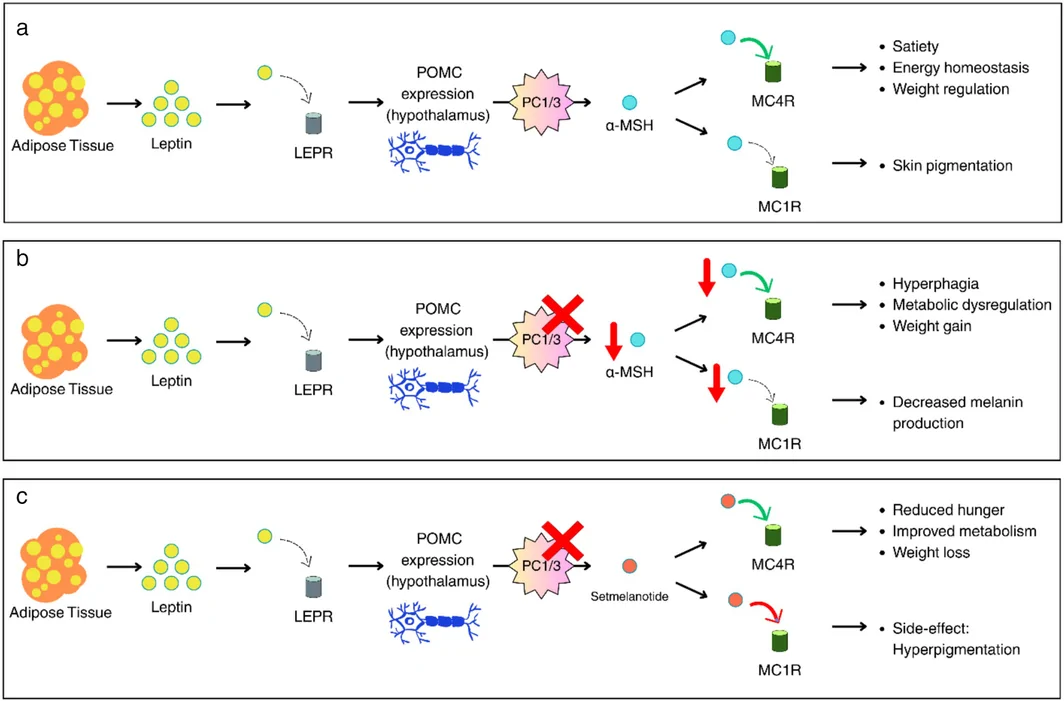
The role of proprotein convertase 1/3 (PC1/3) and setmelanotide's mechanism of action in the MC4R pathway. (a) Physiological PC1/3 function. (b) Impaired catalytic activity of PC1/3. (c) Impaired PC1/3 function with setmelanotide intervention. *Source:* Adapted from: Gor P. et al., 2024 [7]. PC1/3 is encoded by *PCSK1*. ɑ‐MSH, alpha‐melanocyte‐stimulating hormone; LEPR, leptin receptor; MC1R, melanocortin 1 receptor; MC4R, melanocortin 4 receptor; POMC, pro‐opiomelanocortin proprotein.

Setmelanotide is a melanocortin 4 receptor (MC4R) agonist that may reestablish MC4R pathway activity, resulting in hunger reduction, decreased caloric intake, and increased resting energy expenditure, leading to weight loss in some types of monogenic and syndromic obesity [6, 7] (Figure 1). It is currently indicated for chronic weight management in adult and pediatric patients ≥ 6 years old with obesity due to variants of *PCSK1*, pro‐opiomelanocortin (*POMC*), and leptin receptor (*LEPR*) that are pathogenic, likely pathogenic, or of uncertain significance [8, 9].

Promising weight loss outcomes demonstrated in clinical trials may come with side effects [10]. Despite setmelanotide having 20‐fold more activity at MC4R, its concurrent albeit weaker activation of the melanocortin 1 receptor (MC1R) leads to increased expression on melanocytes with accumulation of melanin in skin independently of ultraviolet light [8]. Although clinical trials for setmelanotide reported cutaneous adverse events experienced by patients with *POMC* and *LEPR* deficiency, there is no reported data regarding pigmentation‐related side effects in *PCSK1* mutations [10, 11].

We report a case of a patient diagnosed with a heterozygous *PCSK1*‐p.N221D (rs6232) variant who responded to setmelanotide treatment with weight loss exceeding 11% of her baseline weight. While clinical trials examined treatment effectiveness in the context of complete deficiency in *LEPR* and *POMC* mutations, this is the first case highlighting the effectiveness of setmelanotide in heterozygous *PCSK1* cases, suggesting its strong potential in the setting of partial loss of enzymatic function [3, 10]. We are also the first to report the cutaneous adverse effect of setmelanotide, severe skin hyperpigmentation, in a pathogenic *PCSK1* variant. We also outline the longitudinal clinical course and treatment of our patient, thus emphasizing the need for prompt genetic testing in the context of unclear presentations with early‐onset obesity, hyperphagia, and suboptimal response to conventional weight loss therapies and bariatric surgery [12].

## 2 Case Description

A Caucasian female presented in 2010 at age 40 for preoperative assessment for bariatric surgery consideration. Her ongoing health concerns included obesity, accompanied by significant challenges with losing weight despite multiple attempts utilizing diet, exercise, and group support. Her weight was 116.6 kg with body mass index (BMI) of 40.6 kg/m^2^. A retrospective review of her history revealed that she had been of excess weight since age 6, although she had an unclear history of hyperphagia and described mild to moderate childhood weight gain. There were no reported childhood illnesses, but the presence or absence of chronic gastrointestinal symptoms at those ages was not queried. Physical examination was unremarkable. Her pertinent comorbidities included prediabetes, dyslipidemia, gastroesophageal reflux disease, and well‐managed depression and anxiety. She subsequently met criteria for type 2 diabetes mellitus (T2DM) in 2016. Her family history included obesity, T2DM, and hypertension. Notably, her mother had a history of metastatic melanoma. Laboratory results revealed elevated total cholesterol, low‐density lipoprotein (LDL), triglycerides, glycated hemoglobin (HbA1C), and reduced high‐density lipoprotein (HDL), while creatinine, thyroid, and liver function tests were unremarkable. Proinsulin levels were not measured.

**FIGURE 2:**
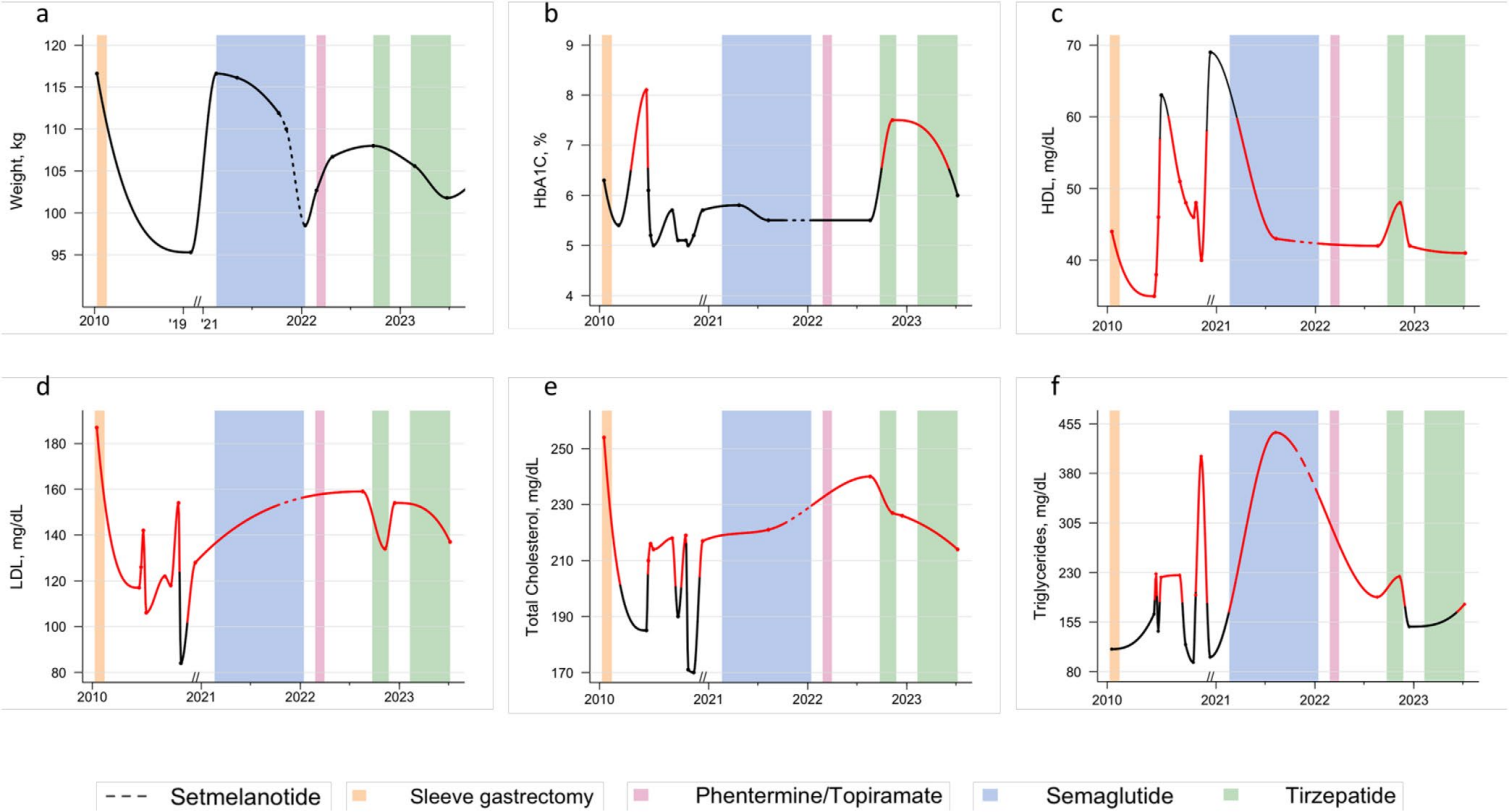
Changes in (a) weight, (b) HbA1C, (c) HDL, (d) LDL, (e) total cholesterol, and (f) triglycerides over the course of the patient's treatment (2010–2023).

Sleeve gastrectomy in 2010 led to a nadir weight of 95 kg (BMI 33.9 kg/m^2^) in 2019, with a total weight loss (%TWL) of 18.6% and excess weight loss (%EWL) of 48.4%, followed by weight regain to baseline levels in the next 2 years (Figure 2a). The dynamics of her weight and laboratory parameters throughout her treatment (2010–2023) are presented in Figure 2a–f.

With semaglutide treatment over 8 months, our patient had 4.3% TWL and 11.0% EWL, although she denied improvements in satiety or cravings (Figure 2a, Table 1). Given her suboptimal responsiveness to lifestyle modifications, pharmacotherapy, and bariatric surgery, and considering her early‐onset obesity, she was offered to undergo genetic testing in 2021. A high‐risk pathogenic heterozygous p.Asn221Asp *PCSK1* mutation was discovered, after which she initiated a trial of setmelanotide 2 mg daily, titrated to 3 mg daily. She continued treatment for ~3 months and experienced significant improvement related to satiety, cravings, and eating control. She lost 13.2 kg, successfully achieving an 11.8% TWL and 32.7% EWL within that time (Figure 2a, Table 1).

**TABLE 1:** Antiobesity pharmacological agents trialed by the patient and their dosages, duration of treatment, effectiveness, and side effects.

Medication	Dosage regimen	Treatment duration, weeks	Weight change, kg	TWL, %	EWL, %	Subjective benefits	Side effects
Semaglutide^a^	0.25 → 1.0 mg weekly (titrated)	33	↓ 5.0	↓ 4.3	↓ 11.0	Unclear benefit on satiety and cravings	None
Setmelanotide^b^	2 mg → 3 mg daily (titrated)	14	↓ 13.2	↓ 11.8	↓ 32.7	Significant improvement in satiety and cravings control	Hyperpigmentation, injection site reactions, mild pruritus, constipation, and nausea
Phentermine/Topiramate	8 mg/25 mg → 8 mg/50 mg daily (titrated)	4–5	↑ 2.3	↑ 2.2	↑ 7.3	None	Insomnia exacerbation
Tirzepatide	2.5 mg → 7.5 mg weekly (titrated)	28–30	↓ 6.4	↓ 5.9	↓ 17.3	Not reported	Anxiety and depression exacerbation and nausea

Abbreviations: EWL, excess weight loss; TWL, total weight loss.

^a^ Semaglutide monotherapy. It was discontinued due to a supply shortage and well‐managed diabetes.

^b^ Setmelanotide treatment overlapped with aemaglutide treatment, and weight loss is reflective of both medications (Figure 2a).

Regarding side effects, she initially experienced mild hyperpigmentation, most noticeable on her face, within 4 weeks of starting setmelanotide. Although there were no new nevi, there was considerable darkening of preexisting nevi, which worsened over time. Dermatologic biopsies obtained due to the patient's family history of melanoma indicated benign skin changes. Due to continued exacerbation, the patient opted to discontinue setmelanotide after 3 months despite significant weight loss benefits. Other side effects included mild pruritus, mild constipation, and injection site reactions. Systemically, there was no worsening in liver or renal functions, HbA1C, or lipid profile (Figure 2b–f).

After setmelanotide, the patient received a short course of phentermine/topiramate, which she did not respond to, exhibiting a 2.2% total weight gain alongside an increase in her HbA1C (Figure 2b). Afterward, she was treated with tirzepatide for 7 months, during which she had 5.9% TWL and 17.3% EWL. As a result of exacerbated depression and anxiety, the medication was discontinued (Figure 2a).

## 3 Discussion

Given our patient's unclear history of hyperphagia and lack of neuroendocrine concerns commonly linked to *PCSK1* mutations, the contribution of a heterozygous p.Asn221Asp variant to her obesity was challenging to predict clinically [12]. In vitro experiments show that the N221D variant results in a 10% to 30% reduction in enzymatic function, while a complete deficiency can result in a reduction of 98.7%, suggesting that there may be significant residual enzymatic function in our patient's case [3, 13, 14]. In terms of function, the N221D construct supports normal maturation and secretion and exhibits unchanged enzyme kinetics, despite reduced activity; however, it may be more vulnerable to environmental stress with decreased stability in certain conditions [13, 14]. This variant has also been shown to confer an average increase of 0.03 BMI units per copy, which potentially translates to a modest BMI increase in the clinical setting [4]. Insulin processing, through measured proinsulin levels, was not assessed in our patient, which is a limitation. In other cases of heterozygous *PCSK1* mutations, proinsulin conversion was normal [15]. Similarly, documentation of gastrointestinal symptoms during the neonatal period or early childhood would provide some evidence for this polymorphism's pathogenicity.

Despite only a 10% to 30% reduction reported in in vitro enzymatic function, there was clear evidence of setmelanotide's effectiveness in our patient, underscoring the value of treatment in a patient with a heterozygous *PCSK1* mutation [3]. She experienced 11.8% TWL and 32.7% EWL in 3 months and reported substantial improvements in eating control, satiety, and cravings. Notably, her treatment with setmelanotide overlapped with semaglutide, which potentially also contributed to such a degree of weight loss; however, the patient reported no significant benefits in satiety on semaglutide monotherapy prior to treatment with setmelanotide.

Our patient's weight loss outcomes with 11.8% TWL are consistent with those seen in clinical trials, with the magnitude of weight loss even exceeding that which might be attributed to concomitant semaglutide treatment. Farooqi et al. reported a 6.9% reduction in BMI in patients with heterozygous variants in *PCSK1, POMC*, or *LEPR* after 6 months of setmelanotide treatment [11]. Studies focusing on other genetic obesity disorders have found a similar benefit of 7.0% weight loss after setmelanotide treatment [11, 16, 17]. Our patient's improvement in satiety, control over eating, and reduced cravings is similar to patients with *POMC* and *LEPR* deficiency who experienced a 27% and 44% decrease in their hunger scores, respectively [10].

To our knowledge, this is also the first documented case of skin hyperpigmentation as a result of setmelanotide in a patient with a pathogenic *PCSK1* variant, as clinical trials have only reported cutaneous side effects in patients with *POMC* and *LEPR* deficiency [10]. Our patient opted to discontinue treatment due to the progressive nature and severity of darkening preexisting melanocytic nevi. Although setmelanotide primarily targets MC4R, thus reestablishing the MC4R pathway activity, there is also some activation of MC1R, which can lead to excessive melanin production and skin hyperpigmentation [7, 8]. In clinical trials, this side effect was reported in all of the *POMC*‐deficient patients and 36% of *LEPR*‐deficient patients, although it has not been reported in any *PCSK1*‐deficient patients [10]. Due to a family history of metastatic melanoma, an oncologic workup in our patient was reasonable; however, it is not routinely necessary [9]. Notably, the pathophysiological pathway of hyperpigmentation as a result of setmelanotide use substantially differs from that of melanoma development, where somatic mutations drive melanocyte proliferation and cause dysregulated melanogenesis [18]. Although the MC1R pathway can be involved, multiple other mechanisms lead to hyperpigmentation in melanoma [18].

Early‐onset obesity, usually with hyperphagia, is a distinctive feature of genetic obesity. Despite a lack of a clear history related to these symptoms, genetic testing revealed a high‐risk *PCSK1* mutation in our patient. Therefore, monogenic obesity should not be disregarded in any patient, and genetic testing needs to be considered as an option if a patient does not adequately respond to lifestyle changes, conventional obesity medications, and/or bariatric surgery.

## 4 Conclusion

We report the first case of a patient with a heterozygous variant of the *PCSK1* gene mutation and a partial loss of enzymatic function, who achieved a total weight loss of 11.8% with setmelanotide treatment, despite a previous suboptimal response to bariatric surgery and conventional obesity medications. It is also the first documented case of severe skin hyperpigmentation as a cutaneous adverse effect of setmelanotide in a patient with a pathogenic *PCSK1* variant. Monogenic obesity should be on the radar as a diagnosis for both obesity medicine specialists and primary care physicians, especially among people with early‐onset obesity accompanied by hyperphagia and those refractory to multidisciplinary approaches, conventional obesity medications, and/or bariatric surgery.

## Funding

The authors have nothing to report.

## Ethics Statement

All procedures performed in studies involving human participants were in accordance with the ethical standards of the institutional and/or national research committee and with the 1964 Helsinki Declaration and its later amendments or comparable ethical standards. Signed consent was obtained from the patient included in the study.

## Conflicts of Interest

E.L. has received consulting fees and honoraria from Novo Nordisk and CPD Network. She also received research support from Novo Nordisk and Eli Lilly. A.R. declared no conflicts of interest. E.G. has received advisory and consulting fees from Novo Nordisk, Currax Pharmaceuticals, Gelesis Inc., and B2M Inc. He has also received research support from the Obesity Treatment Foundation (grant no. OTF001), the Litwin IBD Pioneers Program, the Crohn's and Colitis Foundation, Aardvark Therapeutics, and Eli Lilly.

## Data Availability

The data that support the findings of this study are available on request from the corresponding author. The data are not publicly available due to privacy or ethical restrictions.
